# Indoor air pollution and airway health

**DOI:** 10.1016/j.jaci.2024.08.013

**Published:** 2024-08-23

**Authors:** Jared Radbel, Meghan E. Rebuli, Howard Kipen, Emily Brigham

**Affiliations:** a the Division of Pulmonary and Critical Care Medicine, Rutgers Robert Wood Johnson University, New Brunswick;; b the Department of Pediatrics and Center for Environmental Medicine, Asthma and Lung Biology, University of North Carolina, Chapel Hill;; c the Department of Environmental and Occupational Health and Justice, Rutgers University, Piscataway;; d the Division of Respirology, University of British Columbia, Vancouver;; e the Vancouver Coastal Health Research Institute.

**Keywords:** Indoor air pollution, airway, asthma, COPD

## Abstract

Because of the disproportionate amount of time that people spend indoors and the complexities of air pollutant exposures found there, indoor air pollution is a growing concern for airway health. Both infiltration of outdoor air pollution into the indoor space and indoor sources (such as smoke from tobacco products, cooking or heating practices and combustion of associated fuels, and household materials) contribute to unique exposure mixtures. Although there is substantial literature on the chemistry of indoor air pollution, research focused on health effects is only beginning to emerge and remains an important area of need to protect public health. We provide a review of emerging literature spanning the past 3 years and relating indoor air exposures to airway health, with a specific focus on the impact of either individual pollutant exposures or common combustion sources on the lower airways. Factors defining susceptibility and/or vulnerability are reviewed with consideration for priority populations and modifiable risk factors that may be targeted to advance health equity.

Air pollution is a prominent contributor to global morbidity; it is responsible for at least 1 in 5 deaths worldwide.^1–3^ The majority of evidence supporting health effects is derived from ambient (outdoor) pollution research, in which measurement via government monitors, satellite data, and modeling techniques can provide location-specific concentration estimates. However, individuals spend the vast majority of their time (up to 90%) indoors, where air pollution is neither systematically monitored nor regulated.^4–8^ Studies that aim to detect the health effects of indoor exposures are time and resource intensive, owing to the need to perform individualized assessments. Despite these obstacles, a growing recognition of the importance of research on indoor air quality and health has supported an expanding literature base.

Indoor air is a complex mixture of pollutants determined by local ambient air pollution, degree of infiltration of ambient air into the indoor space (depending on building envelope [Table I]), and contributions of indoor sourcew. Common anthropogenic sources of ambient air pollution in urban centers include industrial and traffic-related pollution, whereas in more rural regions agricultural dust may contribute. Although a broad literature base has focused on indoor biomass burning as a dominant source of indoor air pollution (IAP) in rural regions and/or low- and middle-income countries (LMICs) with associated health risks,^9,10^ attention to the contributions of other myriad indoor sources in industrialized settings is increasing. These indoor sources include combustion processes (cooking, heating, and tobacco product use), building materials and furnishings, and household cleaning and/or personal care products and in-home activities (cleaning, hobbies). Further, over time with the increased frequency and intensity of wildfires,^11^ biomass smoke exposure and infiltration into indoor spaces have become more urgent health considerations even in industrialized settings.

Herein, we will review emerging evidence linking IAP exposure(s) to lower airway disease. This focus is prompted by several considerations. First, the respiratory system serves as a primary site of harm from inhaled exposures. Second, among noninfectious respiratory diseases, processes affecting the airways rank among the highest causes of global mortality and contribute significantly to morbidity.^12,13^ Global asthma and chronic obstructive pulmonary disease (COPD) prevalence are each estimated at approximaely 10%.^14,15^ *In vitro* and *in vivo* mechanisitic studies of lower airway disease or studies of upper airway disease with results for which the biologic mechanisms may be relevant to evolving lower airway processes were also included. Less prevalent airway diseases (eg, obliterative bronchiolitis, cystic fibrosis) and upper airway diseases were not systematically examined owing to limitations in scope; however, mechanistic data specific to airways may have parallel applicability, and expanding relevant literature in these diseases is acknowledged.^16–18^ Finally, as bioaerosols and interventions and/or trials are addressed in other reviews in this series, evidence regarding these aspects of indoor air is not included here.

An understanding of pollutants in the indoor space, source, and health impacts is paramount to protecting population respiratory health. Herein, we review the health effects of common single pollutants measured indoors in the context of those with and without preexisting airway disease. We also discuss health effects linked to specific common sources that are representative of more complex mixtures. Given the substantial literature and recent review of evidence regarding indoor biomass combustion (ie, household air pollution) and lung health in LMICs, we will focus instead on recent literature in developed and/or high-income countries.^10^ Finally, we will discuss specific factors beyond existing respiratory disease that contribute to heightened susceptibility (higher health risk as a result of exposure) and/or vulnerability (higher risk of exposure), with consideration for effects on health equity.

## METHODOLOGY

For this narrative review, we completed an initial search in PubMed using the terms *indoor pollution* and *lung or airway*, with a focus on primary articles published within the past 3 years (2021–2024). To frame the results and conclusions from our 3-year search, we utilized previously published reviews (including a recent review of IAP and airway disease from 2020)^19^ to incorporate seminal studies in the literature published before 2021 along with targeted, supplemental searches to identify relevant articles for subsections of predetermined interest.

## COMMON POLLUTANTS MEASURED IN THE INDOOR ENVIRONMENT AND AIRWAY HEALTH PM

Particulate matter (PM) is a combination of solid and/or liquid particles that are ubiquitous in both outdoor and indoor environments and vary in composition and size. PM size determines regional deposition in the lungs.^20^ Whereas larger particles (ie, coarse PM or PM smaller than 2.5–10 μm [PM_2.5–10_]) deposit largely in the upper airway, smaller particles (ie, fine PM or PM_2.5_) travel deeper into the conducting and respiratory airways and reach the alveoli. The smallest fraction of PM (ie, ultrafine particles or PM_<0.1_) can translocate across the alveolar-capillary interface and enter the systemic circulation, carrying adsorbed toxicants and chemicals.^21^ PM sources in the indoor environment include combustion processes (eg, cooking, heating, candles, incense, tobacco smoke), dust (including biologic contaminants), cleaning, personal care, and some scented products, printers and copiers, and activities that generate dust or use chemicals or glues and adhesives.^22^ Common sources of ambient PM include traffic-related air pollution, industrial and agricultural processes, and wildfire smoke, all of which infiltrate indoor spaces variably, depending on building envelope integrity and inhabitant behaviors. Variability in sources contributes to a diverse composition of PM in the indoor environment, with constituents or adsorbed toxicants, including heavy metals^23,24^ (eg, cadmium, arsenic, lead, zinc, iron), volatile organic compounds (VOCs) and semi-VOCs (including polyaromatic hydrocarbons^23,25–27^ and phthalates [to be discussed further later in this review]),^28,29^ flame retardants^30^ and other organic compounds^31–34^ (including polychlorinated biphenyls [PCBs]), perfluorochemicals,^35^ bioaerosols (discussed separately in another article in this series), and microplastics.^36–38^

Research into the mechanisms by which PM affects airway health has long been centered on inflammatory and oxidative stress pathways^39,40^ influencing cellular function and gene and/or protein expression,^41^ with the occurrence of potential unique processes based on adsorbed toxicants, varied composition, and/or susceptibility.^42^ Indeed, environmentally persistent free radicals can exist on the surface of PM for months, plausibly mediating toxicity.^43^ Studies published within the past 3 years have reinforced or increased recognition of the consequences of PM exposure within *in vitro* models (eg, airway mucosa and alveolar). These consequences may include cilium-related and mitochondrial dysfunction, genotoxicity, morphologic and structural damage to cells, and decreased barrier function.^44–47^ Decreased barrier function is also thought to occur within the lung vascular endothelium, both as a result of direct particle translocation and related toxicity and as a result of inflammation due to PM deposits.^48^ Several additional novel mechanisms have been described in recent years; they include reduced transcript expression and protein levels of a cell membrane channel involved in regulation of sputum viscosity and mucociliary clearance (the cystic fibrosis transmembrane conductance regulator) and impaired defense from viral infection in bronchial epithelial cell culture models following PM exposure.^47,49^

Recent *in vivo* work has highlighted potential outsized effects from indoor particulates on airway health. For example, indoor PM_2.5_ collected in Sweden over the winter months and tested for toxicity in mice via intratracheal installation resulted in a magnified neutrophilic response compared with the response to installation of outdoor PM_2.5_ samples.^50^ Of note, indoor air samples were found to have higher concentrations of polyaromatic hydrocarbons, possibly related to indoor combustion sources. Additionally, higher black carbon content in airway macrophages was associated with lower lung function and more symptoms among current and former smokers with COPD; the associations between indoor PM_2.5_ and black carbon persisted after adjustment for cigarette smoke indicators, likely reflecting indoor air exposures more broadly.^51^

Many recent studies examining the effects of indoor PM focus on susceptible and/or vulnerable groups (highlighted in the relevant section later in this review), reinforcing the diverse negative health consequences associated with elevations in indoor PM, including airway symptoms (wheeze, cough), reduced lung function, asthma- and COPD-related morbidity and mortality, and respiratory infections.^19^ Epidemiologic studies conducted within the past 3 years among adult populations in high-income countries, in which biomass burning is not the dominant fuel source, add to the evidence base of associations between indoor PM concentrations and outcomes related to airway disease. For example, higher concentrations of indoor PM_2.5_ were linked with accelerated lung function decline in former smokers in the United States,^52^ and long-term (10-year) exposure of healthy adults to mean indoor PM_2.5_ concentrations higher than 10 μg/m^3^ has been associated with lower peak flow.^53^ Among individuals living with asthma or COPD, indoor PM exposures (with the most evidence for PM_2.5_) have been associated with reductions in lung function,^54^ and indoor PM_2.5_ has previously been associated with increased symptoms, use of rescue medications, and/or exacerbations.^55,56^ A review of the efficacy of interventions to reduce indoor PM (eg, indoor high-efficiency particle air [HEPA] filtration) is the focus of another article in this series, providing additional insights on the relationship between PM, airway disease, and susceptibility.

### NO_2_

Nitrogen dioxide (NO_2_) is produced by high-temperature combustion from gas stoves, unvented gas heaters, kerosene heaters, gas dryers, and hot water heaters.^57–59^ As NO_2_ has low water solubility, it can penetrate deep into the lung, potentially contributing to lower airway disease.^40^ Exposure to elevated levels of NO_2_ results in free radical–mediated and oxidative stress–induced death of airway cells.^60^ NO_2_ exposure also sensitizes the airways to allergens and contributes to excessive inflammation.^57^ NO_2_ may also translocate directly to the bloodstream, resulting in the formation of methemoglobin.^61^

Previous evidence suggests that prenatal NO_2_ exposure is associated with altered DNA methylation patterns in many mitochondrial and antioxidant genes and that similar epigenetic changes are important in the development of asthma.^40^ Recent evidence from *in vivo* research suggests that pregnant mice exposed to NO_2_ (2.5 ppm for 5 hours per day) exhibit delayed alveolarization of the lungs associated with epigenetic changes, such as differentially expressed long noncoding RNAs and decreased *Sirt1* and *Cxcr2* expression.^62^ Adding to evidence of NO_2_-mediated inflammation, a recent translational Danish study found that 2-year average NO_2_ exposure correlated with plasma levels of the proinflammatory cytokines IL-6 and IFN-γ in adults.^63^

Previous evidence suggests that exposure to NO_2_ is associated with increased respiratory symptoms and lower lung function in infants, children, and adults and is particularly hazardous in people with preexisting asthma.^59,64–70^ More recent studies have reinforced these potential sources of harm. For example, a modeling study of adult current and former smokers without COPD predicted NO_2_ elevations to be derived mainly from cooking while also finding that outdoor levels of NO_2_ had a strong association with indoor NO_2_ levels, thus highlighting the complexity of NO_2_ source contributions.^71^ An additional analysis of this cohort demonstrated a nonsignificant trend toward greater FEV_1_ value decline in former smokers exposed to elevated indoor NO_2_ levels.^52^ With respect to the effects of NO_2_ exposure on asthma development, Chen et al performed a meta-analysis of all population-based or original or review articles on epidemiologic studies of the health effects of air pollutants that were published between 1980 and 2019. Although not specifically addressing the source of NO_2_ (indoor vs outdoor), their random effects model demonstrated a significantly increased risk of childhood asthma with each 10 μg/m^3^-increase in exposure to NO_2_ (relative risk = 1.134 [95% CI = 1.084–1.186] [*P* = .01]).^72^

### O_3_

Ozone (O_3_) is generated by the reaction of oxides of nitrogen and VOCs.^73^ Indoor O_3_ concentrations can reach 20% to 80% of outdoor O_3_ concentrations.^40^ Although indoor O_3_ typically derives largely from outdoor air, indoor sources include photocopiers, certain types of air purifiers, laser printers, and some disinfecting devices (eg, germicidal UV light).^40^ Some germicidal UV systems produce large amounts of (indoor) O_3_ that can additionally drive production of significant amounts of indoor PM_2.5_,^74,75^ Similar to NO_2_, O_3_ is able to penetrate deep into the lung and has irritating effects on the airways that cause cough, increased mucus secretion, and bronchial hyperresponsiveness.^40^ O_3_ contributes to both oxidative stress and inflammation in the airways (damaging the airway epithelium), and early physiologic studies also demonstrated impact on the parasympathetic nervous system esulting in increased pulmonary resistance.^76,77^

Recent translational human studies have helped to elucidate mechanisms of indoor O_3_ oxidative stress–mediated lung toxicity. A study of children with asthma found that personal exposure to indoor O_3_ was associated with increased levels of urinary 6-sulfatoxymelatonin, a surrogate for circulating melatonin, thus suggesting that melatonin is an O_3_-upregulated antioxidant.^78^ Children exposed to elevated levels of O_3_ in classrooms exhibited decreased urine 4-methylumbelliferone levels and increased airway inflammation, as measured by fractional excretion of nitric oxide (Feno), thus suggesting loss of antioxidant buffering from glutathione peroxidase.^79^

Traditionally, studies examining the effects of O_3_ pollution on health outcomes have investigated ambient but not indoor exposure; more recent studies examining indoor O_3_ exposures and impacts on airway disease are few and present mixed results. The previously described systematic review by Chen et al did not observe a reliable and consistent association between O_3_ exposure and respiratory health outcomes; notably, the analysis was not designed to distinguish indoor from outdoor sources of O_3_ exposure.^72^ A cross-sectional study of preschool children in Taiwan measured concentrations of O_3_ inside children’s homes and found no correlation between indoor O_3_ levels and self-reported asthma.^80^ Of note, the mean indoor O_3_ concentrations met Taiwan’s indoor air quality standards and were lower than outdoor O_3_ concentrations.^80^ Alternatively, a study specifically examining indoor O_3_ exposure during sleep in 91 adults aged 18 to 28 years with median measurements of 23.9 μg/m^3^ (interquartile range = 29.5) found decreased forced vital capacity (FVC) values, FEV_1_ values, ratios of FEV_1_ value to FVC, and FEF_25–75_ values for each increase in interquartile range of O_3_ exposure levels.^79^

O_3_ may also interact with other air pollutants with complex effects. For example, as discussed further later in this review, VOCs are prevalent in indoor air at concentrations that are orders of magnitude greater than in outdoor air.^81^ Many of these VOCs, particularly those with carbon-carbon double bonds, readily undergo oxidation by indoor O_3_ to form, among other chemical species, secondary organic aerosol, which is a major component of PM_2.5_.^81^ O_3_ is the driver of this VOC-dependent chemistry whose reaction products have recently been associated with increased levels of Feno and other biomarkers of airway inflammation.^82^

### VOCs

VOCs are chemicals that evaporate under normal atmospheric conditions.^83^ They can be further classified as very volatile VOCs (propane, butane, methyl chloride), volatile VOCs (formaldehyde, d-limonene, toluene, acetone, ethanol), and semi-VOCs (pesticides such as dichlorodiphenyltrichloroethane [DDT] and chlordane, plasticizers [phthalates], and fire retardants such as polychlorinated biphenyls and polybrominated biphenyls [PBBs]),^83^ and they can be aliphatic or aromatic compounds. They are ubiquitous in virtually all indoor environments owing to their prevalence as intentional or unintentional components of products used in construction, including paints, glues, adhesives, processed wood products such as flooring, and synthetic carpets. An attached garage may also be an important source of VOCs for the adjacent residential space. Other important sources include activities and products used in daily life, such as tobacco smoke, cleaning products, air fresheners and other scented products, natural gas combustion, and cooking.^84^ Indoor concentrations typically exceed outdoor concentrations by a range of magnitudes from 2- to 10-fold.^83^

VOCs react directly with the airway epithelium,^85,86^ although some studies suggest that oxidative stress as well as sensitization remain important elements of VOC airway toxicity.^87,88^ Recent *in vitro* and *in vivo* studies have helped elucidate mechanisms of VOC airway toxicity. A respiratory cell line–based study found that the flame retardant triphenyl phosphate, a semivolatile indoor pollutant, induced dose-dependent cytotoxicity, increased reactive oxygen species (ROS) production, and altered antioxidant production.^89^ Further, particularly in the human leukemia factor 1 (HLF1) and human nasal epithelial cell (HNEpC) cell lines, exposure to flame retardant also induced endoplasmic reticulum stress and altered mitochondrial function and immune mediator production.^89^ Exposure of Flp-In 293 cell lines to 2-ethyl-hexanol and texanol increased expression of transient receptor potential ankyrin 1 (TRPA1), a previously described inducer of bronchial hyperresponsiveness.^90^ After exposure to formaldehyde, *ex vivo* alveolar cells from rat lungs express rno_circRNA_006061, which binds to rno-miR-128–3p, coregulating p38/ATF3-mediated apoptosis.^91^

Prior animal model evidence suggests that phthalates contribute to airway remodeling by stimulating migration and proliferation of bronchial cells.^92^ In a recent ovalbumin-induced mouse model, flame retardants (tri-n-butyl phosphate and tris (2-butoxythyl) phosphate) were found to induce respiratory bronchial muscle and goblet cell hypertrophy and exacerbate respiratory inflammation and ovalbumin-stimulated asthmatic responses, indicating pulmonary toxicity through the nuclear factor-kB (NF-kB) pathway.^93^ A recent translational clinical trial found T-cell lines to be altered following inhalation challenge with dibutyl phthalate, with increased numbers of circulating CD4^+^ T_H_ cells and decreased numbers of regulatory T cells.^94^

Over the previous decades, many epidemiologic studies have addressed the role of total indoor VOCs in general or that of specific agents in causing or exacerbating airway diseases such as asthma or COPD, with emerging evidence indicating that these VOCs and agents are a prenatal risk factor for pulmonary abnormalities later in life.^95,96^ Recent evidence supporting the idea that VOCs are a risk factor for the development of airway disease is mixed. Since 2021, there have been 4 meta-analyses of the association between exposure to total or specific VOCs and asthma onset and/or symptoms.^54,85,86,97–99^ Holtjer et al reviewed 75 prior reviews of observational evidence for risk factors for adult-onset asthma and/or COPD.^86^ VOCs in general, as well as formaldehyde specifically, were found to be risk factors for adult asthma but were not identified as risk factors for COPD.^86,100,101^ In a further meta-analysis of cohort, case-control, or cross-sectional studies reporting dose-response data excluding formaldehyde exposures, Liu et al reported significant pooled increased risk for asthma for every 1-μg/m^3^ increase in concentrations of benzene, toluene, paradichlorobenzene, and tetrachloroethylene.^97^ In another recent meta-analysis that was limited largely to high-income countries and covered studies that included empirical data and results in the form of coefficients (including odds ratios, relative risk, and correlation), Alford et al found that total VOCs had a statistically significant effect size (Cohen disease) of 0.37 (medium strength) for new-onset asthma.^85^ Although formaldehyde has long been considered a cause of irritant exacerbator of airway disease, one recent review argued that much of the epidemiologic data supporting this association may be confounded by concomitant exposure to acrolein.^102^

Despite these positive meta-analyses, the overall weight of evidence for VOCs as a cause for asthma is not as strong as one might think. A recent high-quality systematic review from the European Academy of Allergy and Clinical Immunology that included observational studies or experimental studies assessing children or adults with or without asthma, found only very weak support for VOCs as a cause of asthma in children or adults.^103^ These findings are in accord with those of a well-conducted meta-analysis of observational and intervention studies that reported antenatal and postnatal exposures and outcomes in children and adults and similarly concluded that the epidemiologic database for asthma incidence from home exposure to VOCs is very weak.^96^ The seeming discrepancy between these findings and those of the 4 aforementioed meta-analyses may be explained by the fact that the review by Agache et al did not include phthalates in its analysis.^103^

Regarding phthalates specifically, in a study of slightly fewer than 400 adults without preexisting respiratory disease, dermal skin wipes used to measure phthalate exposure indicated an association of exposure with altered lung function; higher dermal phthalate levels were associated with lower FEV_1_ and FVC values in elderly participants.^104^ Whether the lung function deficits were related to inhalation, dermal absorption, or both is not clear. In a recent study using a model of controlled exposure followed by bronchoalveolar wash and lavage, dibutyl phthalate was examined as a potential adjuvant or exacerbator in allergensensitized individuals. Exposure before allergen challenge increased the early allergic response (as measured by decline in FEV_1_ area under the curve), increased airway hyperresponsiveness in those without baseline hyperresponsiveness, and altered the degree of cellular response (macrophage recruitment) in the airways.^105^ The importance of phthalates in asthma onset and severity is further supported by a recent epidemiologic study from Taiwan in which patients with asthma were found to have higher urinary levels of a variety of phthalates than those of the controls and total phthalate levels were associated with increased risks of emergency room visits and hospitalization.^106^ Thus, recent evidence suggests that among VOCs, indoor phthalate exposure in particular may contribute to airway disease.

### Radon

Long-term exposure to radon gas has traditionally been linked with incident lung cancer^107^; however, it may also contribute to airway disease. Radon is formed from the natural decay of uranium in the soil; it migrates from geologic sources to accumulate in indoor spaces as well as in drinking water. Although more recent studies have also linked radon exposure to changes in lung function and airway disease, neither *in vitro* nor *in vivo* studies on this topic have been completed.

Emerging literature suggests a role of radon in decrements in lung function and airway disease. A 2022 study of primarily elderly males with COPD in Eastern Massachusetts demonstrated that reduced FEV_1_ and FVC values are linked with exposure to higher concentrations of radon decay products attached to airborne particles.^108^ Alternatively, in a recent study of individuals with COPD in Spain, there was no association between exposure radon in concentrations of 600 Bq/m^3^ or less and lung function.^109^ A study of another Spanish cohort examined the association between radon exposure and COPD as well as intersection with smoking; in the overall study population no association was noted between radon exposure (measured in the home among individuals in the same dwelling living for ≥15 years) and COPD onset; however, a significant relationship emerged among smokers.^110^ Additional epidemiologic and mechanistic studies are needed to better elucidate the relationship between radon exposure and airway disease.

## COMMON SOURCES OF MIXED IAP AND AIRWAY HEALTH

### Tobacco products, smoke, and aerosols

The dangers of tobacco smoke generated through the combustion of the tobacco plant and inhaled via cigarettes, cigars, or cigarillos include a variety of respiratory health impacts, including COPD and lung cancer. Although exposure to tobacco smoke is highest among those actively smoking, secondhand and thirdhand smoke exposure (environmental tobacco smoke) in indoor spaces also affects respiratory health.^111^ Tobacco smoke contains a complex mixture of particulates, gases, and associated chemicals that are directly toxic to the airway epithelium.

Whereas both cigarette smoke and exposure to biomass burning have been documented to contribute to COPD, deposition of inhaled smoke in lower airway generations may explain the increases in emphysema in cigarette smoke–exposed individuals versus in those exposed to biomass smoke, as identified via *in silico* analyses.^112^ Another recently proposed mechanism of tobacco-induced lung disease identified in an *in vitro* study demonstrates the role of circular RNAs in response to cigarette smoke and induction of emphysema, suggesting that cigarette smoke–induced decreases in specific circular RNA levels may alter microRNA regulation of gene expression and explaining the development of cigarette smoke–induced emphysema.^113^

The emergence of e-cigarettes, which are electronically heated devices that contain a mixture of humectants, nicotine, and flavoring compounds, has sparked additional concerns. Although the long-term effects of e-cigarette use are not yet known owing to the limited time of their use in the population, short-term effects have been documented; these effects include respiratory outcomes such as e-cigarette– or vaping-associated lung disease, wheeze, and shortness of breath.^114^ Although firsthand exposure can be attributed to a variety of respiratory outcomes, secondhand and thirdhand exposure to aerosols and related outcomes are currently understudied. Research examining the effects of mechanisms in *in vivo* or *in vitro* models are especially lacking.

Environmental tobacco smoke exposure increases the risk of respiratory symptoms, rhinitis symptoms, wheeze, and nocturnal cough.^115^ It has also historically been linked to decrements in lung function. However, when cessation is achieved, lung function parameters have been noted to improve not only among ever smokers but also among never smokers in the population.^116^ Recent studies demonstrate that environmental tobacco smoke can also alter responses to other inhaled IAP exposures. In one study using this modeling paradigm, cigarette smokers with altered morphologic and physiologic parameters have been found to receive lung doses of radon progeny smaller than those received by nonsmokers, although the differrence amounted to less than 15%.^117^ However, increased doses were found in extrathoracic organs.^117^ Although current smokers did not demonstrate an association between indoor PM concentration and lung function decline, former smokers with COPD living in indoor spaces with higher PM concentrations demonstrated accelerated loss of lung function compared with the loss of function seen in those with lower PM concentrations, suggesting that both smoking cessation and indoor air quality may be important modifiers of lung function decline.^52^ In this analysis, never smokers were not available for comparison. An additional review found that having COPD due to smoking increases susceptibility to indoor biomass combustion and wildfire smoke.^118^ Recently, secondhand exposure has been shown to contribute to bronchitis symptoms and shortness of breath among a general population of young adults recruited from entire classrooms.^119^ Other new and emerging products of concern that contribute to IAP include combusted and aerosolized cannabinoid products and inhaled nutraceuticals and aromatherapy products (eg, “wellness” vapes); the effects of these products were not included in the results of the search criteria used for this review.^120^

### Cooking with gas stoves

Cooking processes in general, and in particular, those occurring at high temperatures and involving oil, fat, and charring processes, routinely contribute to IAP (including peaks in indoor PM concentrations).^121^ Cooking with gas stoves further contributes to IAP by producing combustion-related increases in levels of PM, O_3_, and nitrogen oxides.^122^ Herein, we will focus on IAP generated from cooking with gas stoves as a pollutant mixture.

In our search, we found recent *in vitro* and *in vivo* work examining mechanisms of cooking fume–related lung disease. Exposure of human epithelial cells to gas cook stove extracts increased gene expression within ferroptosis and NRF2 oxidative stress pathways that was associated with methylation and hypomethylation of key genes within these pathways, suggesting altered epigenetic regulation.^123^ In *in vivo* research, female rats exposed intratracheally to cooking oil fumes (although not specifically from gas stoves) once every 3 days for 30 days exhibited airway obstruction with eosinophilia, as well as increased levels of markers of oxidative stress (increased malondialdehyde and 8-OhdG levels but decreased glutathione levels) and markers of apoptosis (caspase 3 and NF-κB), inflammation (TNF-α and IL1-β) in lung homogenates, and endoplasmic reticulum stress (IRE-1α and caspase-12) by immunohistochemistry of lung tissue.^124,125^

Although cooking with gas has previously been linked in literature to asthma in children, its effects on airway disease in adults are unclear.^126^ Our search continued to find evidence of an association between gas stove use and airway disease in children (described later). In adult populations, there were 2 new studies examining the association of cooking with gas and airway disease and yielding mixed results. An adjusted analysis of a large multi-center European cohort of randomly selected adults aged 20 to 44 years from the general population found increased self-reported shortness of breath at rest with gas stove use versus with electric stove use, although a diagnosis of airway disease was not specifically examined.^127^ A multivariate analysis of a Danish cohort that included an examination of questions regarding gas stove use did not find a significant association between use of a gas stove and FEV_1_ value decline.^63^

### Wood smoke and biomass smoke from wildfire

Indoor wood smoke in high-income, developed nations may be encountered in the setting of wood stove or pellet stove heating (most often in rural regions) or home fireplace use. Although much of the fuel for wildfires is wood (the smoke from which can travel thousands of miles and intrude into indoor spaces, thereby affecting indoor air quality),^128^ combustion of additional biomass materials in forests (ie, vegetation) and materials at the urban-wildland interface (eg, infrastructure [including buildings and vehicles]) contributes to a complex mixture of air pollutants that has traditionally been referred to as biomass smoke rather than wood smoke from wildfires. Biomass smoke from wildfires further undergoes photochemical aging during travel from the source, contributing to an increasingly complex mixture of pollutants that enter indoor spaces and affect indoor air quality. Both biomass smoke from wildfires and wood smoke contain a combination of PM, carbon and nitrogen oxides, VOCs, and polyaromatic hydrocarbons, as well as numerous other compounds dependent on source and aging.^129^ Wood smoke is sometimes used as a proxy for biomass smoke owing to wildfires in experimental studies.

Several *in vitro* and animal studies have examined health effects of exposure to wood smoke and biomass smoke, providing clues as to health effects that may be observed with indoor exposure in future human studies. Collectively, animal and *in vitro* studies identify inflammation, oxidative stress, mitochondrial dysfunction, impaired response to viral infection, and altered airway cellular function as common mechanisms of response to exposure to wildfire and wood smoke.^130,131^ A recent *in vitro* investigation of wood smoke exposure in primary bronchial epithelial cells identified increases in ROS levels, increased NF-κB levels, and altered gene expression with enriched mitochondrial dysfunction, inflammation, oxidative stress, chronic respiratory disorder, and ciliary-related pathways, among other things.^46^ Within animal models, exposure of guinea pigs to wood smoke results in upregulation of matrix metalloproteinases, tissue inhibitors of metalloproteinases, and inflammatory cytokines (specifically, TGF-β, TNF-α, IFN-ɣ, IL-1β, IL-6, and IL-8) in lung tissue.^132^

Supporting the evidence of the potential harm of exposure found in the aforementioned *in vitro* and *in vivo* studies, a recent longitudinal study in Australia examined the health impacts of exposure to prolonged wildfire smoke in 240 people during the 2019–2020 bushfire period.^133^ Exposure was associated with respiratory symptoms (breathlessness, wheeze and/or whistling in the chest, and cough), asthma exacerbation, and reduced capacity to participate in usual activities.^133^ Symptoms were not sufficiently mitigated by staying indoors with windows and doors shut, and asthma symptoms persisted in 65% of subjects following the bushfire period.^133^ An additional study examined the effects of wildfire smoke on early childhood respiratory health; the study found increases in the use of respiratory medication with exposure in the early postnatal period.^134^ On the basis of these recent studies and reviews and reports, it can be stated that wildfire and biomass smoke have the potential to detrimentally affect the airway and other biologic systems and exposure should be avoided or reduced as much as possible in indoor spaces.^135–137^

## FACTORS CONTRIBUTING TO VULNERABILITY AND/OR SUSCEPTIBILITY

**FIG 1.**
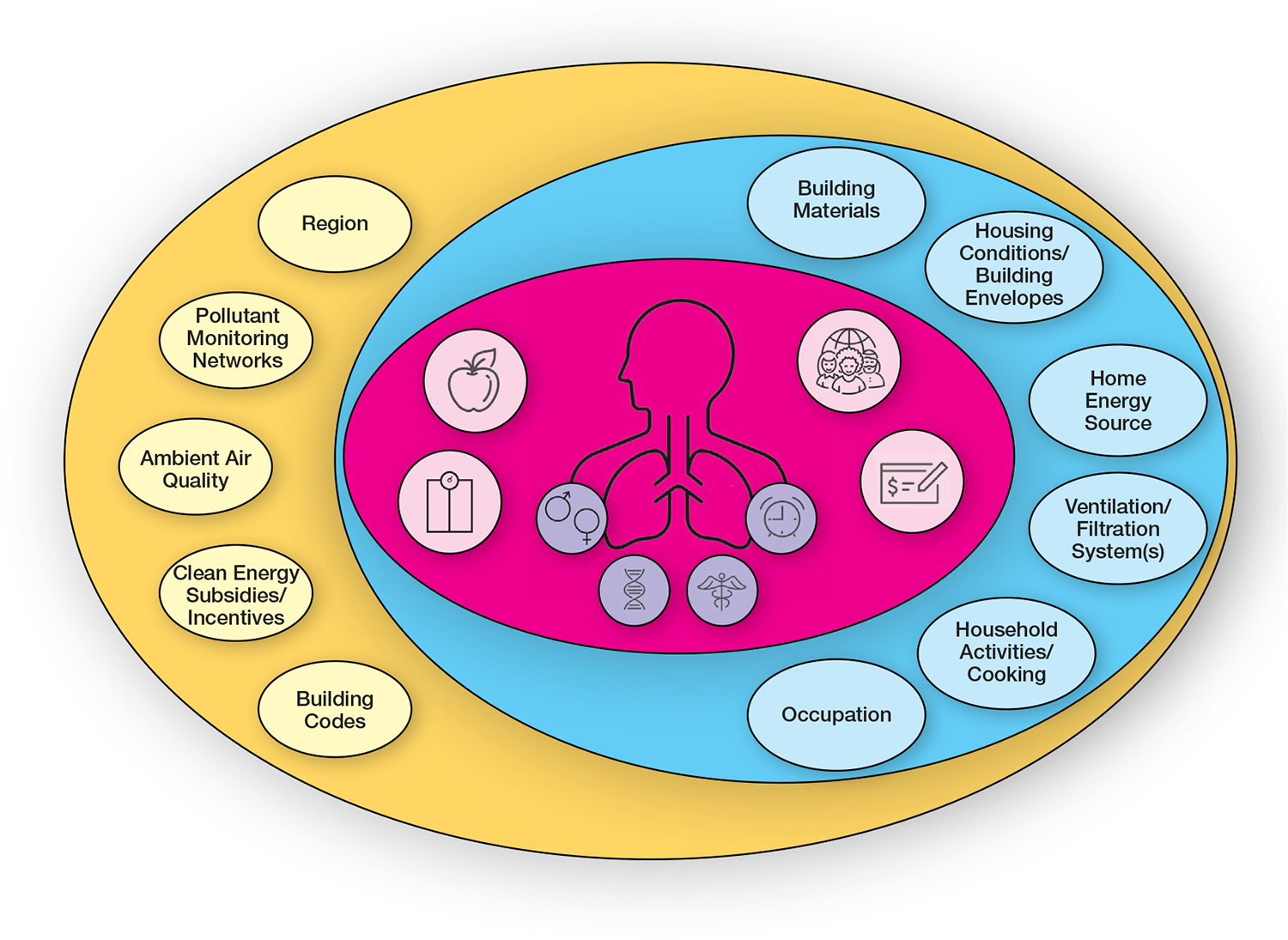
Considerations for susceptibility and/or vulnerability to IAP. Schematic representing many of the factors linked to susceptibility and/or vulnerability to indoor air, organized within 3 domains: within-individual (*blue oval*); individual-level environment (*green oval*; and local, regional, and national environment (*orange oval*). Within-individual factors include intrinsic biologic factors (*purple circles indicate sex, genetics, health conditions, and age*) and personal factors linked strongly with behaviors and socioeconomic status (*blue circles indicate body mass index and/or adiposity, and diet, race, and/or ethnicity income and/or socioeconomic status*). Factors that influence the biologic response determine susceptibility, whereas factors that influence the degree of exposure determine vulnerability.

Many of the studies noted in the aforementioned summarized evidence focus on populations with preexisting respiratory disease given recognized susceptibility. However, as within any population and indeed within any individual, multiple factors converge to confer higher or lower vulnerability (higher likelihood of exposure or exposure to higher concentrations) or susceptibility (biologic effect at a given concentration) (Fig 1).

### Socioeconomics

In addition to the previously reviewed studies in LMICs, there has been recent work examining vulnerability to IAP based on socioeconomic status in middle- to high-income countries. Similar to the work done in LMICs, a systematic review of fuel poverty studies including data from France, the United Kingdom, Germany, Bulgaria, Russia, Romania, and the United States found that fuel poverty in low-income homes can increase the risk of asthma and that energy-efficient improvements in low-income housing can improve respiratory symptoms.^138^ An English modeling study found a linear increase in household PM_2.5_ exposure with decreasing household income as well as increased PM_2.5_ exposure in schools located in deprived areas.^139^

### Race/ethnicity

Studies examining racial and ethnic disparities in air pollution exposure focus largely on outdoor concentrations, in which case there is evidence of disproportionately higher exposures (conferring vulnerability) among Black, Hispanic, and Asian populations than among White populations.^140^ Given penetration of outdoor pollutants into the indoor environment, this may contribute to disparities in IAP and resultant airway health impacts; however, this question remains underexplored.^141^ Further, differential housing and housing conditions,^142^ as well as different product choices and rates of behaviors potentially occurring in individual and neighboring indoor spaces (eg, personal care product use and tobacco product use)^143,144^ may further contribute to differential indoor air exposures, but they remain understudied.

### Age (infancy and childhood)

Infants and children are a population routinely hypothesized to have potential enhanced susceptibility to IAP. This hypothesis stems from ongoing immune and respiratory system development and growth during infancy and childhood.^54^ Additionally, with a higher minute ventilation in relation to body mass, infants and children (as compared with adults) may receive a higher effective dose of inhaled pollutants at the same concentration.^145^ Studies comparing susceptibility of IAP by age were not found, likely owing to the sample size necessary for effect modification analyses, and this represents an unmet need in IAP research.

Emphasizing the impact of IAP in child and/or youth populations, 2 recent original investigations (1 narrative review and 1 meta-analysis) examined the link between gas stove use and pediatric asthma. A large analysis of data from US children aged 2 to 16 years found that children from homes that used ventilation while operating gas stoves were less likely to be diagnosed with asthma, chronic bronchitis, or report wheeze.^122^ When effect sizes from meta-analysis, including studies from North America and Europe, were applied, it was further reported that an impressive 12.7% of childhood asthma in the United States may be attributable to gas stove use.^146^ However, a latent class analysis of a large Danish cohort failed to find a significant association between childhood exposure to gas stoves with adolescent onset asthma.^147^

Children are also susceptible to the effects of VOCs and radon. A study examining the association between phthalate levels found in indoor dust and croup in infants at age 6 months found significant associations with diethylphthalate and diethylhexylphthalate.^28^ When stratified by sex, the male infant associations were greater than the aggregate, whereas the effects in female infants were difficult to estimate owing to a low number of subjects.^28^ Paint, which is known to emit VOCs (including propylene glycol), was studied as a potential contributor to asthma attacks in a population of children with asthma and found to increase the likelihood of an asthma attack versus in children without exposure (odds ratio = 10.49 [95% CI = 1.16–94.85]).^148^ Maung et al performed a systematic review that included a focus on VOC exposures in children and other high-risk groups.^54^ High VOC levels were associated with worsening asthma symptoms.^54^ Children living close to industrial sites had more exposure to VOCs, and there was a significant association with school absence because of sore throat, cough, and cold.^54^ They also noted traffic jams as a novel source of VOC exposure.^54^ One study also reported o-xylene from industrial emissions as being associated with increased respiratory symptoms.^54^ Lastly, a study on school-age asthmatic children in the United States found increases in Feno levels, as well as maximum asthma symptom days with radon exposure.^149^

There are ample data regarding the impacts of indoor biomass smoke exposures in children in LMICs; for example, a systematic review that included 11 studies of children found an association between IAP and decreased FEV_1_ and FVC values in 6 and 7 studies, respectively, with reductions in FEV_1_ and FVC values specifically attributed to use of biomass cooking in 2 and 4 studies, respectively. No relationship with FEV_1_/FVC ratio was found^150^; however, the available data regarding IAP and airway disease in healthy child populations in high-income countries are limited. Of note, a recent study examining PM_2.5_ and PM_10_ concentrations failed to demonstrate an association with air trapping in a cohort of US children aged 5 to 17 years who were monitored for 6 months.^151^

### Genetics

As evidence regarding the impact of pollutants on airways via inflammatory and oxidative stress pathways mounts, genetic polymorphisms that confer differential individual responses along these pathways are being explored as etiologies of susceptibility; for example, multiple polymorphisms exist for glutathione-S-transferase enzymes (GST), which play a role in detoxification and regulation of oxidative stress.^152^ Recent publication of results from a Tasmanian study of long-term (10 years) household air pollution described an augmented risk of asthma and reduced lung function as a result of pollutant exposures among those with the *GSTP1* Ile/Ile genotype.^153^ The potential importance of GST polymorphisms in determining associations between indoor air pollutants, asthma risk, and lung function was further highlighted by a 2018 systematic review.^154^ These associations are largely mirrored in epidemiologic studies of ambient air pollution in which genes in oxidative and nitrosative stress pathways have been implicated in susceptibility,^155^ and the associations are further mirrored in controlled exposure studies.^156,157^ In a cohort exposed to dibutyl phthalate, peroxisome proliferator–activated receptor-γ (*PPARG*) genetic variants were also found to modulate airway and systemic immune responses, including allergen-specific levels of IgE in blood, T_H_2 cells in blood, and T_H_2 cells in bronchoalveolar lavage fluid.^158^ Lastly, a recent review found that single-nucleotide polymorphisms in heat shock protein 90 gene complex (*HSP90B1*), Hedgehog-interacting protein (*HHIP*), and α-1 antitrypsin (*A1AI*) genes confer differing risk of COPD development related to cigarette smoking and biomass exposures.^39^

### Sex and gender

Evidence regarding differential susceptibility to the effects of IAP by sex or gender in higher-income countries is lacking. Differential exposures by sex or gender may also drive vulnerability; to date, however, they have not been systematically explored with regard to IAP in high-income countries. Other potential mechanisms of susceptibility include sex-specific expression of detoxifying enzymes in target tissues such as the lung,^159^ disruption of secreted hormones or binding to hormone receptors by air pollutants affecting endocrine regulation of airway immune health, and sex chromosome contributions to pollutant detoxification/clearance in the lungs and development of environmental lung disease.^160–162^

### Diet and overweight/obesity

Several prior studies suggest that poor diet and overweight or obesity may enhance susceptibility to IAP with regard to airway disease.^163–165^ Diets low in antioxidant intake and high in proinflammatory factors may augment the respiratory response to other proinflammatory/oxidant exposures, including IAP.^166^ Obesity, which has been associated with incident and prevalent airway disease,^167^ alters airway mechanics, which plausibly affects pollutant deposition and response.^168^ Further, obesity is also associated with heightened systemic inflammation and oxidative stress, which similar to poor diet, may plausibly reduce resilience to additional proinflammatory/oxidant exposures.^169^ Interestingly, air pollution has also been posited as a potential contributor to the development of obesity, with described mechanisms including inflammation and oxidative stress as well as epigenetic changes and alteration of the gut microbiome.^170^

### Conclusions and research needs

The current review highlights the continued evolution of knowledge and growing body of research characterizing the impact of IAP on airway disease. At the present time, research provides mechanistic and epidemiologic evidence of a relationship between higher IAP and airway disease development, lung function decline, respiratory symptoms, and exacerbation of preexisting airway disease. Of note, detecting impact among healthy populations versus among populations that include those with preexisting disease often requires higher sample size and subclinical markers of physiologic and biologic change. We have been careful to note these differences in study population where defined, as extrapolation of effects between populations with and without disease may not be valid. Further diversity in exposure classification and outcomes measured and reported across studies presents a challenge in evidence review, limiting summative conclusions. With a focus on emerging literature building on prior research, we have noted several specific evidence gaps that provide an opportunity for research to advance fundamental understanding of the effects of IAP on airway disease (Table II). These include but are not limited to stratification of data by sex and gender, investigation of potential racial disparities in IAP, interactions between pollutants in the indoor space and impact on health, and additional biologic factors conferring susceptibility that may foster individualized interventions. Investment in research infrastructure is of growing importance given the escalating need to optimize indoor air while also advancing health equity in the context of a changing climate.

## Figures and Tables

**TABLE I.** Factors affecting infiltration of outdoor air to the indoor environment

Infrastructure	Inhabitant behaviors
Walls, foundation, roof construction	Building maintenance
Window and door seals	Window/door time open vs time shut
Heating/cooling systems	Recirculation mode use
Ventilation system	Changing/application of filters

Structural components that provide a barrier between indoor and outdoor environments are known as a building envelope. Initial construction and maintenance/upkeep can both affect the integrity of the envelope, as can behaviors of inhabitants (window and door closure, ventilation settings, filtration), ultimately affecting the contribution of outdoor air pollution to indoor air exposures.

**TABLE II.** Gaps in knowledge and emerging issues in IAP and airway health in high-income and developed countries

Understudied and/or emerging exposures in IAP research
Radon gas Wildfire smoke infiltration Combusted and aerosolized cannabinoid products Inhaled nutraceuticals and aromatherapy products
Mechanisms of IAP-related airway disease
Radon Mixed pollutants
IAP exposure models
Indoor vs outdoor O_3_ Permeation of pollution in newer vs older construction Mixed IAP exposures Gas vs electric stoves
Populations at increased risk from IAP
Sources of socioeconomic disparities of health related to IAP Relative harm based on sex and gender Unidentified genetic susceptibility Comparisons of IAP-related health outcomes across the lifespan

Several gaps in knowledge present an opportunity for research to advance the fundamental understanding of the effects of IAP on airway disease. These gaps can be broken down into insufficient knowledge regarding mechanisms of pollutant injury, understudied associations between certain indoor air pollutants and airway disease, need for more advanced exposure models, and elucidation of populations at increased risk of harm from IAP.

## Abbreviations used

COPD: Chronic obstructive pulmonary disease
Feno: Fractional exhaled nitric oxide
FVC: Forced vital capacity
GST: Glutathione-S-transferase
HNEpC: Human nasal epithelial cell
IAP: Indoor air pollution
LMIC: Low- and middle-income country
NF-κB: Nuclear factor-κB
NO_2_: Nitrogen dioxide
O_3_: Ozone
PM: Particulate matter
ROS: Reactive oxygen species
RR: Relative risk
VOC: Volatile organic compound
